# Association of dietary intake with cancer of the digestive system: a cross-sectional study

**DOI:** 10.3389/fnut.2025.1539401

**Published:** 2025-01-22

**Authors:** Xinxin Qin, Litao Ge, Song Wu, Wei Li

**Affiliations:** Department of Gastrocolorectal Surgery, General Surgery Center, The First Hospital of Jilin University, Changchun, China

**Keywords:** dietary factors, NHANES, gastric cancer, liver cancer, colorectal cancer, nutrients

## Abstract

**Background:**

In recent years, the incidence of cancers of the digestive system has been increasing, posing a severe threat to the lives and health of people around the world, and has become one of the leading causes of cancer deaths worldwide. The three most common cancers of the digestive system include gastric, colorectal, and liver cancers, and attention has been paid to the role of diet in the progression of these cancers. However, the relationship between dietary factors and cancers of the digestive system remains to be investigated.

**Methods:**

This study included 30,789 adults aged 20 years or older from the National Health and Nutrition Examination Survey (NHANES), conducted from 2007 to 2018. It assessed the association between 30 dietary factors and digestive system cancers. Descriptive analysis was used to explore the demographic characteristics of the participants and *p*-values were calculated using a weighted linear regression model. Categorical variables were described as percentages, and *p*-values were calculated using weighted chi-square tests.

**Results:**

We found that protein, vitamin B1, calcium, and iron intake were positively associated with colorectal cancer; vitamin B2 and phosphorus intake were negatively related to colorectal cancer; dietary folate and vitamin B12 intake were negatively associated with gastric cancer; vitamin D and copper intake were positively associated with gastric cancer; vitamin E intake was negatively related to the development of hepatocellular carcinoma; and lycopene, vitamin B2, calcium, iron, and zinc intake was positively associated with the development of liver cancer. Other than that, we did not observe any correlation between other dietary factors and cancers of the digestive system.

**Conclusion:**

Dietary intake is associated with digestive system cancers, and more epidemiologic studies are needed to validate our results.

## 1 Introduction

The incidence of digestive tumors is increasing year by year throughout the world. It is increasingly constituting a public health problem that threatens the world’s health and safety. Gastric, colorectal, and liver cancers are common digestive system tumors. Gastric cancer is the fourth leading cause of cancer deaths worldwide and the fifth most common cancer (1). Colorectal cancer, on the other hand, is the third most common malignancy and the second deadliest cancer globally. In contrast, the increase in the incidence of colorectal cancer is mainly attributed to the increased exposure to environmental risk factors due to westernization of lifestyles and diets (2–4). Although the therapeutic outlook for colorectal cancer is generally favorable, the increasing number of patients with colorectal cancer and the rising trend of younger incidence still pose a heavy economic burden and a significant public health challenge globally (3, 5, 6). A previous study of a U.S. population showed that people with high frequency and daily alcohol consumption had a greatly increased risk of colorectal cancer (7). The research of Li’s team suggests that heavy drinking, smoking and a sedentary lifestyle all increase the risk of colorectal cancer (8). A case–control study also elaborated that dairy consumption, obesity and red meat consumption increased the risk of colorectal cancer (9). Primary liver cancer, on the other hand, is the seventh most common cancer in the world and the second leading cause of cancer-related deaths globally (10). Primary liver cancer includes hepatocellular carcinoma, intrahepatic cholangiocarcinoma, and other types of liver cancer. They account for about 80, 15, and 5%, respectively (11). In 2018, 21.5% of gastrointestinal cancer cases worldwide were linked to dietary factors (12). To date, the relationship between dietary intake and cancers such as colorectal cancer, breast cancer, prostate cancer, and lung cancer has been extensively studied (13–16).

People have begun to examine the relationship between dietary intake and cancers of the digestive system in recent years. A pooled analysis of 14 prospective studies concluded that fruit and vegetable intake was not strongly associated with a reduced risk of colon cancer overall, but may be associated with a reduced risk of distal colon cancer (17). The World Cancer Research Fund/American Institute for Cancer Research (WCRF/AICR) panel, in its Continuing Update Project (CUP), concluded that the evidence that foods high in dietary fiber protect against colorectal cancer and that consumption of red meat, processed meats, and alcohol (primarily in men) increases the risk of colorectal cancer is “compelling.” Milk and calcium may be associated with a lower risk of colorectal cancer, while there is limited evidence that folic acid, selenium, and vitamin D may be protective (18). Although there were significant differences in population characteristics, study design, and methodology across studies, plant-based diets (with some dairy and fish) were associated with a lower risk of colorectal cancer, whereas diets high in meat, refined grains, and added sugars appeared to increase risk (19–22). A prospective study found that alpha-carotene can effectively reduce the risk of liver cancer (23). A previous prospective study found that dietary fiber and coffee intake were negatively associated with developing hepatocellular carcinoma. In contrast, dietary glycemic index, sugar-sweetened beverages, and trans fats were positively related to the development of hepatocellular carcinoma (24). Different dietary factors have been associated with a higher risk of gastric cancer, such as low intake of fruits and vegetables, high intake of red and processed meat and preserved foods, and inadequate intake of several antioxidant minerals and vitamins (25–30).

Changes in diet structure often cause compensatory changes in other nutrients because the human body’s demand for nutrients is multifaceted; when the intake of a certain nutrient changes, it may affect the intake and utilization of other nutrients (31). Focusing on food groups or eating patterns allows for a more comprehensive assessment of the impact of diet on health. This approach takes into account the interactions between foods and overall dietary structure, rather than individual nutrients. By analyzing food groups or dietary patterns, researchers can avoid accidental associations due to nutrient covariation and more accurately assess the health effects of diet (32). Therefore, by including multiple dietary factors, this study can provide a comprehensive view of the potential relationship between diet and digestive system tumors.

The National Health and Nutrition Examination Survey (NHANES) is a research program led by the U.S. Centers for Disease Control and Prevention (CDC) to assess the health of adults and children nationwide (33). The study was designed to examine the relationship between dietary intake and cancer of the digestive system using a representative sample of Americans.

## 2 Methods

### 2.1 Study design and population

The population sample for this study was extracted from the NHANES database, a research program designed to assess the health and nutritional status of adults and children in the U.S. The survey is unique in that it combines interviews and physical examinations. It selected a representative noninstitutionalized U.S. population. Participants were interviewed at home, followed by various clinical and laboratory tests at a mobile examination center (MEC). The survey collected comprehensive data on demographics, socioeconomic status, diet and health. All the results of the survey have been weighted (34). We combined six consecutive NHANES surveys from 2007–2008, 2009–2010, 2011–2012, 2013–2014, 2015–2016, and 2017–2018 into one analytic sample.30789 adults aged 20 years or older were interviewed about dietary intake and medical conditions. NHANES was reviewed and approved by the NCHS Ethics Review Board, and all participants provided written informed consent by the Declaration of Helsinki.

### 2.2 Outcomes

The primary endpoint of the study was the diagnosis of cancer of the digestive tract. Cancer type was defined based on items on a medical status questionnaire, “Have you ever been told by a doctor or other health professional that you have cancer or a malignant tumor?” and “What type of cancer?” Answers indicating only stomach, colorectal, and liver cancers were categorized as outcome variables.

### 2.3 Dietary intakes

Trained interviewers conducted two consecutive 24-h dietary recalls to assess total nutritional intake by comprehensively referencing the NHANES. The first was conducted face-to-face during the MEC exam, and the second was collected by telephone 3–10 days later. Dietary intake was calculated from the average of the two dietary recall data (if available); otherwise, single nutritional recall data were used.

We incorporated 30 dietary factors from the dietary questionnaire in the NHANES database. These factors contained four nutrients, 11 vitamins, four carotenoids and nine minerals, including protein (g), total sugars (g), total fat (g), cholesterol (mg), vitamin A (vitamin A, RAE(Retinol Activity Equivalents)) (ug), alpha-carotene (ug), beta-carotene (ug), beta-cryptoxanthin (ug), lycopene (ug), vitamin B1 (thiamine) (mg), vitamin B2 (riboflavin) (mg), niacin (mg), vitamin B6 (mg), dietary folate (ug), vitamin B12 (ug), vitamin C (ug), vitamin D (D2 + D3) (ug), vitamin E as alpha-tocopherol (mg), vitamin K (ug), calcium (mg), Phosphorus (mg), Magnesium (mg), Iron (mg), Zinc (mg), Copper (mg), Sodium (mg), Potassium (mg) and Selenium (mcg) as well as Caffeine (mg) and Alcohol (g).

### 2.4 Covariates

We included age, gender, race, energy intake (expressed in kilocalories), body mass index (BMI), diabetes mellitus, hypertension, poverty-to-income ratio (PIR), education level, and smoking as covariates in this study.

### 2.5 Statistical analysis

Continuous variables were described using descriptive analyses, and *p* values were calculated using weighted linear regression models. Categorical variables were described using percentages, and *p* values were calculated using weighted chi-square tests. Logistic regression analyses were performed using STATA software, and p values <0.05 were considered statistically significant. In calculating the weights, we divided the two-year cycle weights by six to reflect the six survey cycles.

## 3 Results

### 3.1 Characteristics of included participants

**Figure 1:**
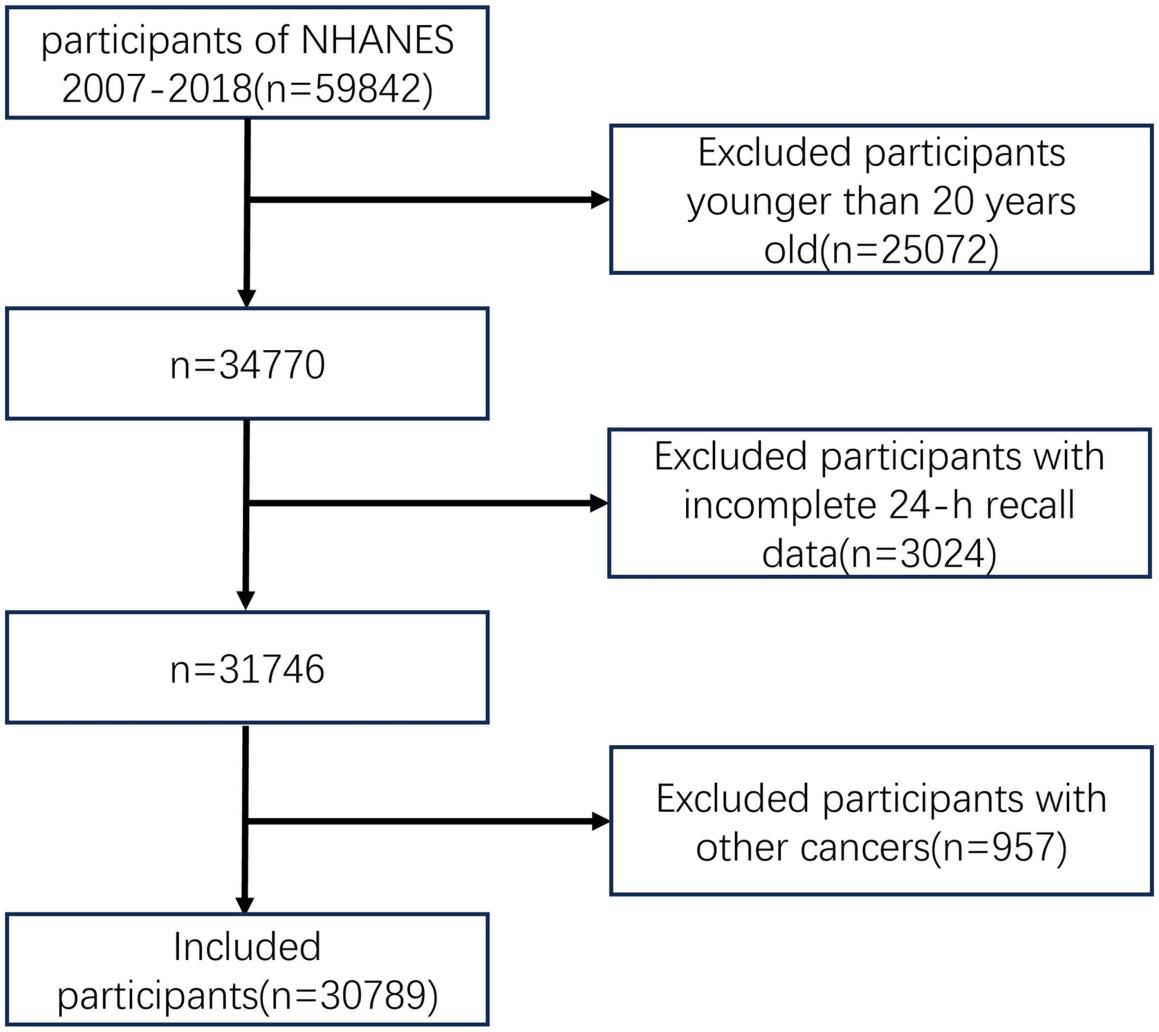
Flowchart of study participants.

The flow chart of this study is shown in Figure 1. Compared with participants without cancer, colorectal cancer patients were older (*p* < 0.001), tended to be Non-Hispanic White (*p* < 0.001), and were predominately smokers (*p* = 0.007). Patients with gastric cancer were older (*p* < 0.001), tended to have higher levels of education (*p* = 0.003), and were predominantly smokers (*p* = 0.024). Patients with liver cancer were older (*p* < 0.001), had less daily energy intake (*p* = 0.013), and had higher BMI (*p* = 0.003). The baseline characteristics of participants in this study are shown in Table 1.

**Table 1:** Characteristics of participants with and without self-reported gastric cancer, colorectal cancer and liver cancer, separately.

Variables		Colorectal cancer			Gastric cancer			Liver cancer		
	Total (*N* = 30,789)	No (*N* = 30,563)	Yes (*N* = 226)	*p* value	No (*N* = 30,748)	Yes (*N* = 41)	*p*- value	No (*N* = 30,756)	Yes (*N* = 33)	*p* value
Age (years)	47.51 ± 16.97	47.39 ± 16.92	67.73 ± 13.08	<0.001	47.49 ± 16.96	66.49 ± 13.11	<0.001	47.50 ± 16.97	63.13 ± 10.91	<0.001
Energy (kcal)	2101.65 ± 856.07	2103.35 ± 856.78	1812.32 ± 664.07	<0.001	2101.90 ± 855.98	1816.02 ± 909.55	0.082	2102.02 ± 856.20	1699.73 ± 569.62	0.013
Gender				0.117			0.671			0.156
Men	48.26 (47.50–49.02)	48.30 (47.53–49.06)	42.42 (34.18–51.11)		48.26 (47.50–49.02)	52.33 (33.23–70.78)		48.27 (47.51–49.03)	34.91 (16.53–59.22)	
Female	51.74 (50.98–52.50)	51.70 (50.94–52.47)	57.58 (48.89–65.82)		51.74 (50.98–52.50)	47.67 (29.22–66.77)		51.73 (50.97–52.49)	65.09 (40.78–83.47)	
Race				<0.001			0.611			0.750
Mexican American	8.44 (8.16–8.73)	8.48 (8.20–8.77)	1.99 (0.99–3.99)		8.45 (8.16–8.74)	1.58 (0.38–6.44)		8.45 (8.16–8.74)	3.47 (0.94–11.98)	
Other Hispanic	5.79 (5.56–6.03)	5.81 (5.57–6.05)	2.29 (1.24–4.19)		5.79 (5.56–6.03)	5.99 (1.17–25.62)		5.79 (5.56–6.03)	3.25 (0.88–11.26)	
Non-Hispanic White	66.90 (66.29–67.50)	66.81 (66.20–67.42)	81.97 (76.67–86.28)		66.89 (66.28–67.50)	73.21 (56.53–85.17)		66.90 (66.29–67.50)	67.58 (46.11–83.55)	
Non-Hispanic Black	11.19 (10.88–11.50)	11.20 (10.89–11.51)	9.02 (6.42–12.53)		11.18 (10.88–11.50)	15.53 (7.85–28.39)		11.18 (10.88–11.50)	14.07 (6.43–28.05)	
Other Race - Including Multi-Racial	7.68 (7.35–8.03)	7.70 (7.37–8.05)	4.73 (2.54–8.64)		7.69 (7.35–8.04)	3.68 (0.90–13.95)		7.68 (7.35–8.03)	11.64 (3.18–34.53)	
Education				0.492			0.033			0.916
Less than 9th grade	5.19 (4.97–5.43)	5.18 (4.96–5.42)	6.66 (4.19–10.43)		5.19 (4.96–5.42)	11.65 (4.40–27.42)		5.19 (4.97–5.43)	4.07 (1.34–11.65)	
9th–11th grade	10.43 (10.05–10.83)	10.42 (10.04–10.82)	12.07 (8.37–17.12)		10.42 (10.04–10.82)	22.15 (11.22–39.05)		10.43 (10.05–10.83)	12.40 (5.12–27.09)	
More than 12th grade	84.37 (83.92–84.82)	84.39 (83.94–84.84)	81.27 (75.37–86.02)		84.39(83.94–84.84)	66.20 (47.93–80.65)		84.38 (83.92–84.82)	83.53 (68.04–92.35)	
Family Poverty-Income-Ratio				0.257			0.097			0.708
0–1	13.61 (13.20–14.03)	13.62 (13.20–14.04)	12.14 (8.22–17.56)		13.61 (13.20–14.03)	12.24 (4.87–27.52)		13.61 (13.19–14.03)	16.17 (7.15–32.60)	
1–2	26.20 (25.59–26.81)	26.18 (25.57–26.79)	29.88 (22.97–37.86)		26.18 (25.57–26.79)	47.50 (29.48–66.19)		26.21 (25.60–26.82)	18.68 (7.86–38.20)	
2–3	26.30 (25.63–26.99)	26.28 (25.60–26.96)	31.03 (23.17–40.16)		26.30 (25.63–26.99)	26.00 (11.23–49.41)		26.30 (25.63–26.98)	32.52 (14.29–58.20)	
3–4	9.68 (9.19–10.19)	9.69 (9.20–10.20)	7.67 (4.19–13.63)		9.68 (9.19–10.19)	2.84 (0.62–12.03)		9.67 (9.18–10.18)	14.22 (2.40–52.73)	
>4	24.21 (23.49–24.95)	24.24 (23.52–24.98)	19.28 (12.76–28.07)		24.22 (23.50–24.96)	11.42 (2.91–35.65)		24.22 (23.50–24.95)	18.41 (4.92–49.61)	
Diabetes				<0.001			0.002			0.693
Yes	9.84 (9.43–10.27)	9.73 (9.32–10.15)	29.08 (22.22–37.06)		9.82 (9.41–10.25)	27.76 (13.85–47.89)		9.84 (9.43–10.26)	12.05 (5.48–24.46)	
No	90.16 (89.73–90.57)	90.27 (89.85–90.68)	70.92 (62.94–77.78)		90.18 (89.75–90.59)	72.24 (52.11–86.15)		90.16 (89.74–90.57)	87.95 (75.54–94.52)	
Smoking				0.007			0.024			0.268
Yes	44.15 (43.40–44.91)	44.09 (43.34–44.85)	54.05 (45.24–62.60)		44.13 (43.38–44.89)	65.62 (43.83–82.36)		44.14 (43.39–44.90)	54.52 (30.43–76.67)	
No	55.85 (55.09–56.60)	55.91 (55.15–56.66)	45.95 (37.40–54.76)		55.87 (55.11–56.62)	34.38 (17.64–56.17)		55.86 (55.10–56.61)	45.48 (23.33–69.57)	
BMI (kg/m2)				0.312			0.007			0.003
<18.5	1.53 (1.36–1.72)	1.54 (1.36–1.73)	0.56 (0.11–2.78)		1.52 (1.35–1.72)	9.21 (3.23–23.58)		1.53 (1.36–1.72)	2.21 (0.30–14.63)	
18.5–24.9	28.30 (27.62–28.99)	28.33 (27.64–29.02)	23.61 (17.22–31.46)		28.29 (27.61–28.98)	34.89 (20.33–52.95)		28.30 (27.61–28.99)	32.44 (13.46–59.71)	
25.0–29.9	32.53 (31.82–33.24)	32.52 (31.81–33.24)	33.63 (25.74–42.55)		32.53 (31.82–33.24)	31.84 (15.57–54.20)		32.50 (31.79–33.22)	59.10 (34.27–80.01)	
≥30.0	37.64 (36.91–38.38)	37.62 (36.88–38.35)	42.20 (33.87–51.00)		37.66 (36.93–38.39)	24.05 (10.89–45.07)		37.67 (36.94–38.41)	6.26 (2.11–17.10)	
Hypertension				<0.001			0.471			0.780
Yes	21.15 (20.55–21.75)	21.05 (20.46–21.66)	37.59 (29.84–46.04)		21.14 (20.55–21.75)	26.80 (14.21–44.74)		21.15 (20.55–21.75)	23.30 (10.11–45.07)	
No	78.85 (78.25–79.45)	78.95 (78.34–79.54)	62.41 (53.96–70.16)		78.86 (78.25–79.45)	73.20 (55.26–85.79)		78.85 (78.25–79.45)	76.70 (54.93–89.89)	

### 3.2 Dietary intakes and digestive system risk

Table 2 lists the dietary intake of the study population in this study. Patients with colorectal cancer consumed less protein (*p* < 0.001), total sugars (*p* = 0.010), fat (*p* = 0.001), vitamin E (*p* = 0.002), lycopene (*p* = 0.008), vitamin B2 (*p* = 0.038), niacin (*p* = 0.001), vitamin B6 (*p* = 0.037), dietary folic acid (*p* < 0.001), vitamin C (*p* = 0.016), calcium (*p* = 0.018), phosphorus (*p* < 0.001), magnesium (*p* < 0.001), copper (*p* = 0.024), sodium (*p* < 0.001), potassium (*p* = 0.016), selenium (*p* < 0.001) and alcohol (*p* = 0.004). In addition, patients with gastric cancer consumed less protein (*p* = 0.009), vitamin B1 (*p* = 0.028), niacin (*p* = 0.003), vitamin B6 (*p* = 0.020), dietary folate (*p* = 0.010), vitamin C (*p* = 0.042), phosphorus (*p* = 0.030), and selenium (*p* = 0.021). Patients with liver cancer consumed less fat (*p* = 0.049), lycopene (*p* = 0.019) and selenium (*p* = 0.041). No relevant statistical differences were observed in other dietary factors in GI tumors (*p* > 0.05).

**Table 2:** Dietary intakes in participants with and without self-reported gastric cancer, colorectal cancer and liver cancer, separately.

Variables	Colorectal cancer			Gastric cancer			Liver cancer		
	No (*N* = 30,563)	Yes (*N* = 226)	*p* value	No (*N* = 30,748)	Yes (*N* = 41)	*p* value	No (*N* = 30,756)	Yes (*N* = 33)	*p*- value
Protein (g)	82.23 ± 36.16	71.78 ± 28.69	<0.001	82.18 ± 36.14	64.08 ± 28.27	0.009	82.18 ± 36.14	69.60 ± 23.16	0.065
Total sugars (g)	109.73 ± 67.22	96.73 ± 64.16	0.010	109.66 ± 67.22	101.43 ± 58.22	0.524	109.67 ± 67.23	93.18 ± 43.02	0.193
Total fat (g)	81.48 ± 40.01	71.82 ± 32.85	0.001	81.43 ± 39.97	77.89 ± 49.18	0.646	81.44 ± 39.99	66.60 ± 28.57	0.049
Cholesterol (mg)	291.15 ± 195.88	250.09 ± 166.29	0.005	290.91 ± 195.71	290.55 ± 230.59	0.993	290.93 ± 195.77	269.76 ± 166.01	0.566
Vitamin E (mg)	8.68 ± 5.91	7.28 ± 4.58	0.002	8.67 ± 5.91	6.98 ± 4.60	0.136	8.67 ± 5.91	6.65 ± 3.31	0.070
Vitamin A (μg)	644.11 ± 576.32	615.06 ± 445.77	0.500	643.99 ± 575.75	586.20 ± 447.17	0.602	644.03 ± 575.84	544.75 ± 282.06	0.360
Alpha-carotene (μg)	427.00 ± 1211.23	418.28 ± 945.06	0.923	427.08 ± 1210.24	283.73 ± 613.65	0.538	426.90 ± 1210.29	484.09 ± 529.81	0.802
Beta-carotene (μg)	2305.39 ± 4134.14	2009.92 ± 2762.32	0.339	2304.28 ± 4128.72	1598.61 ± 2308.58	0.374	2303.84 ± 4128.98	2109.07 ± 1971.27	0.802
Beta-cryptoxanthin (μg)	84.59 ± 186.05	73.98 ± 91.66	0.445	84.53 ± 185.70	77.32 ± 103.45	0.840	84.53 ± 185.69	78.01 ± 118.80	0.852
Lycopene (μg)	5283.31 ± 7623.30	3764.65 ± 5697.83	0.008	5276.36 ± 7616.19	3114.89 ± 4665.17	0.140	5271.37 ± 7605.95	8651.05 ± 13661.80	0.019
Vitamin B1 (mg)	1.62 ± 0.81	1.54 ± 0.73	0.175	1.62 ± 0.81	1.28 ± 0.58	0.028	1.62 ± 0.81	1.64 ± 0.93	0.901
Vitamin B2 (mg)	2.15 ± 1.16	1.97 ± 0.91	0.038	2.15 ± 1.16	1.82 ± 1.04	0.140	2.15 ± 1.16	2.17 ± 1.29	0.951
Niacin (mg)	25.91 ± 13.80	22.34 ± 9.98	0.001	25.90 ± 13.78	17.89 ± 7.83	0.003	25.89 ± 13.78	23.40 ± 11.02	0.337
Vitamin B6 (mg)	2.14 ± 1.48	1.90 ± 1.11	0.037	2.14 ± 1.48	1.47 ± 0.78	0.020	2.14 ± 1.48	1.82 ± 0.94	0.259
Food folate (μg)	220.40 ± 126.16	184.00 ± 82.70	<0.001	220.24 ± 126.00	157.51 ± 88.32	0.010	220.22 ± 126.00	183.80 ± 99.97	0.125
Vitamin B12 (μg)	5.16 ± 5.07	4.86 ± 3.98	0.431	5.16 ± 5.07	3.83 ± 2.57	0.173	5.15 ± 5.07	4.42 ± 2.13	0.439
Vitamin C (μg)	81.58 ± 78.68	67.34 ± 54.19	0.016	81.52 ± 78.57	50.86 ± 70.96	0.042	81.50 ± 78.57	80.51 ± 69.28	0.947
Vitamin D (μg)	4.64 ± 4.59	4.32 ± 3.31	0.354	4.63 ± 4.59	4.52 ± 4.87	0.899	4.63 ± 4.59	3.60 ± 3.48	0.231
Vitamin K (μg)	116.40 ± 230.26	89.70 ± 82.07	0.121	116.28 ± 229.77	75.87 ± 59.83	0.360	116.28 ± 229.77	76.15 ± 81.73	0.354
Calcium (mg)	964.04 ± 516.56	872.49 ± 433.90	0.018	963.64 ± 516.09	803.03 ± 568.11	0.106	963.54 ± 516.24	928.60 ± 427.80	0.720
Phosphorus (mg)	1379.47 ± 592.75	1190.89 ± 453.17	<0.001	1378.59 ± 592.17	1131.58 ± 587.52	0.030	1378.57 ± 592.31	1165.15 ± 414.21	0.056
Magnesium (mg)	301.95 ± 134.37	263.72 ± 106.95	<0.001	301.77 ± 134.24	259.41 ± 146.68	0.101	301.76 ± 134.27	265.47 ± 106.88	0.152
Iron (mg)	14.85 ± 7.71	14.62 ± 7.78	0.689	14.85 ± 7.71	12.82 ± 6.69	0.171	14.85 ± 7.71	16.58 ± 10.83	0.234
Zinc (mg)	11.45 ± 6.42	11.07 ± 6.78	0.430	11.45 ± 6.42	9.13 ± 4.54	0.060	11.44 ± 6.42	12.61 ± 6.15	0.334
Copper (mg)	1.27 ± 0.79	1.13 ± 0.59	0.024	1.27 ± 0.79	1.25 ± 0.97	0.902	1.27 ± 0.79	1.17 ± 0.54	0.511
Sodium (mg)	3489.83 ± 1543.20	3008.41 ± 1149.47	<0.001	3487.41 ± 1541.87	3051.28 ± 1158.56	0.141	3487.50 ± 1541.88	2968.59 ± 1130.06	0.074
Potassium (mg)	2671.78 ± 1110.00	2470.70 ± 955.36	0.016	2670.95 ± 1109.24	2276.13 ± 1067.38	0.064	2670.97 ± 1109.38	2276.29 ± 896.27	0.059
Selenium (μg)	114.13 ± 54.47	98.61 ± 42.90	<0.001	114.06 ± 54.43	89.85 ± 39.10	0.021	114.05 ± 54.43	93.08 ± 43.05	0.041
Caffeine (mg)	167.36 ± 188.59	180.78 ± 168.65	0.342	167.45 ± 188.51	152.35 ± 156.13	0.677	167.40 ± 188.47	205.75 ± 191.83	0.281
Alcohol (g)	9.72 ± 23.05	4.74 ± 15.50	0.004	9.69 ± 23.03	2.02 ± 5.74	0.083	9.69 ± 23.03	3.03 ± 5.58	0.125

After adjusting for potential confounders, the associations between dietary intake and digestive system tumor cancers are shown in Table 3. After adjusting for covariates, protein [OR, 95%CI; 1.024(1.005–1.043), *p* = 0.013], vitaminB1 [OR, 95%CI; 1.480 (1.042–2.102), *p* = 0.029], calcium [OR, 95%CI; 1.001 (1.000–1.002), *p* = 0.001] and iron [OR, 95% CI; 1.042 (1.008–1.078), *p* = 0.015] intake were positively associated with colorectal cancer; vitamin B2 [OR, 95% CI; 0.560 (0.325–0.965), *p* = 0.037] and phosphorus [OR, 95% CI; 0.998 (0.997–1.000), *p* = 0.011] intake were negatively associated with colorectal cancer. Food folate [OR, 95% CI; 0.989 (0.981–0.997), *p* = 0.005] and vitamin B12 [OR, 95% CI; 0.837 (0.703–0.996), *p* = 0.045] intake were negatively associated with gastric cancer; vitamin D [OR, 95% CI; 1.048 (1.014–1.084), *p* = 0.005] and copper [OR, 95%CI; 3.489 (1.245–9.776), *p* = 0.017] intake were positively associated with gastric cancer. In addition, vitamin E [OR, 95% CI; 0.839 (0.741–0.949), *p* = 0.005] intake was negatively associated with liver cancer; lycopene [OR, 95%CI; 1.000 (1.000–1.000), *p* < 0.001], vitamin B2 [OR, 95%CI; 1.657 (1.058–2.595), *p* = 0.027], calcium [OR, 95%CI; 1.002 (1.001–1.003), *p* < 0.001], iron [OR, 95% CI; 1.095 (1.013–1.185), *p* = 0.023] and zinc [OR, 95% CI; 1.090 (1.014–1.171), *p* = 0.019] intake were positively associated with liver cancer.

**Table 3:** ORs with 95% CIs of the associations between dietary intakes, gastric cancer, colorectal cancer and liver cancer, separately.

Variables	Colorectal cancer		Gastric cancer		Liver cancer	
	OR (CI)	*p*- value	OR (CI)	*p*- value	OR (CI)	*p*- value
Protein (g)	1.024 (1.005–1.043)	0.013	0.996 (0.96–1.033)	0.818	1.022 (0.972–1.074)	0.392
Total sugars (g)	1.002 (0.994–1.011)	0.594	1.003 (0.989–1.017)	0.718	1.009 (0.993–1.025)	0.276
Total fat (g)	1.008 (0.989–1.027)	0.436	1.026 (0.998–1.055)	0.069	1.034 (0.979–1.092)	0.229
Cholesterol (mg)	0.999 (0.997–1.001)	0.452	1.002 (0.999–1.005)	0.123	1.003 (0.999–1.007)	0.137
Vitamin E (mg)	0.981 (0.933–1.030)	0.436	0.938 (0.861–1.021)	0.140	0.839 (0.741–0.949)	0.005
Vitamin A (μg)	1.000 (0.999–1.001)	0.832	1.000 (0.998–1.002)	0.978	0.998 (0.995–1.000)	0.066
Alpha-carotene (μg)	1.000 (1.000–1.000)	0.669	1.000 (0.999–1.001)	0.642	1.000 (1.000–1.001)	0.298
Beta-carotene (μg)	1.000 (1.000–1.000)	0.637	1.000 (1.000–1.000)	0.993	1.000 (1.000–1.000)	0.311
Beta-cryptoxanthin (μg)	1.000 (0.999–1.001)	0.470	1.000 (1.000–1.000)	0.096	0.999 (0.997–1.002)	0.595
Lycopene (μg)	1.000 (1.000–1.000)	0.215	1.000 (1.000–1.000)	0.749	1.000 (1.000–1.000)	<0.001
Vitamin B1 (mg)	1.480 (1.042–2.102)	0.029	0.825 (0.308–2.214)	0.703	1.122 (0.606–2.077)	0.714
Vitamin B2 (mg)	0.560 (0.325–0.965)	0.037	1.207 (0.630–2.314)	0.571	1.657 (1.058–2.595)	0.027
Niacin (mg)	0.976 (0.942–1.011)	0.179	0.946 (0.856–1.045)	0.270	1.030 (0.925–1.146)	0.591
Vitamin B6 (mg)	1.128 (0.936–1.361)	0.206	0.826 (0.300–2.278)	0.712	0.884 (0.370–2.114)	0.782
Food folate (μg)	0.999 (0.996–1.002)	0.503	0.989 (0.981–0.997)	0.005	1.003 (0.997–1.008)	0.368
Vitamin B12 (μg)	0.997 (0.967–1.028)	0.842	0.837 (0.703–0.996)	0.045	0.823 (0.650–1.042)	0.106
Vitamin C (μg)	0.997 (0.992–1.001)	0.099	0.994 (0.983–1.005)	0.257	1.002 (0.995–1.010)	0.568
Vitamin D (μg)	0.976 (0.907–1.051)	0.517	1.048 (1.014–1.084)	0.005	0.881 (0.698–1.111)	0.284
Vitamin K (μg)	0.999 (0.996–1.001)	0.249	0.999 (0.994–1.003)	0.553	0.996 (0.989–1.004)	0.366
Calcium (mg)	1.001 (1.000–1.002)	0.001	1.001 (0.998–1.003)	0.668	1.002 (1.001–1.003)	<0.001
Phosphorus (mg)	0.998 (0.997–1.000)	0.011	0.998 (0.995–1.002)	0.337	0.999 (0.996–1.003)	0.774
Magnesium (mg)	0.999 (0.994–1.003)	0.607	1.005 (1.000–1.010)	0.074	1.003 (0.992–1.013)	0.628
Iron (mg)	1.042 (1.008–1.078)	0.015	1.061 (0.996–1.132)	0.067	1.095 (1.013–1.185)	0.023
Zinc (mg)	1.016 (0.989–1.045)	0.250	0.926 (0.840–1.021)	0.121	1.090 (1.014–1.171)	0.019
Copper (mg)	1.024 (0.615–1.706)	0.928	3.489 (1.245–9.776)	0.017*	0.827 (0.256–2.670)	0.750
Sodium (mg)	1.000 (1.000–1.000)	0.566	1.000 (1.000–1.001)	0.076	1.000 (1.000–1.000)	0.943
Potassium (mg)	1.000 (1.000–1.001)	0.137	1.001 (1.000–1.001)	0.227	0.999 (0.997–1.000)	0.064
Selenium (μg)	1.001 (0.990–1.011)	0.917	0.997 (0.989–1.005)	0.429	0.985 (0.962–1.009)	0.220
Caffeine (mg)	1.001 (1.000–1.002)	0.110	0.999 (0.996–1.001)	0.296	1.000 (0.996–1.004)	0.972
Alcohol (g)	1.001 (0.980–1.021)	0.962	0.975 (0.936–1.015)	0.221	0.979 (0.930–1.031)	0.419

The intake of other nutrients, vitamins, and minerals were not correlated with colorectal, gastric, or liver cancer.

## 4 Discussion

In this cross-sectional study, we reveal the relationship between dietary intake and digestive system cancers. To our knowledge, this is the first study to comprehensively examine the relationship between nutritional factors and digestive system cancers in the U.S. population using the NHANES database.

The prospect that intake of specific vitamins may have anticancer effects has received much attention in recent years (35–37). Our study found that vitamin B12 intake was negatively associated with the risk of gastric cancer. This may be attributed to the fact that vitamin B12 deficiency may contribute to cancer pathogenesis by reducing DNA synthesis, leading to altered expression of cancer-related genes (38).

In recent years, the relationship between vitamin D and cancer has received attention from many researchers (39–42). Epidemiologic findings seem to be inconsistent regarding whether vitamin D reduces the incidence of gastric cancer. Several meta-analyses have shown no significant relationship between gastric cancer incidence and vitamin D (43). However, other studies have shown a positive correlation between vitamin D intake and the incidence of gastric cancer, and the results of this paper are similar (44). In contrast, some studies have shown that insufficient vitamin D intake reduces the incidence and mortality of gastric cancer (45). Previous studies’ conflicting conclusions may be attributed to various factors involved in the vitamin D absorption pathway in the body, such as sunlight exposure and various hormones. In this study, food folate intake was negatively associated with stomach cancer. Folic acid plays a vital role in maintaining DNA stability, and if folic acid is deficient, it can cause DNA damage. At this point, inappropriate gene expression is caused by hypomethylation of DNA, which affects DNA repair and causes chromosomal breaks, leading to the development of cancer. Polymorphisms in methylenetetrahydrofolate reductase (MTHFR), a key enzyme in folate metabolism, may also play a role in cancer development (46, 47). In this study, vitamin B2 intake was negatively associated with colorectal cancer. Studies of vitamin B2 and the risk of colorectal carcinogenesis are limited. A prospective study on the role of vitamins in reducing the risk of colorectal cancer development found no evidence that a high intake of vitamin B2 reduces the risk of colorectal cancer (48). Xu’s team found that serum vitamin B2 levels were negatively correlated with the development of colorectal cancer in a study that included 1,009 cases of colorectal cancer patients (49). Interestingly, the results of previous research support the idea that high levels of vitamin B2 may play a role in promoting the development of colorectal cancer (50). Therefore, further studies are needed to investigate whether vitamin B2 intake is effective in preventing the development of colorectal cancer. Vitamin B2 intake was positively related to the development of liver cancer. A recent prospective study found no statistical effect when stratifying the study population by sex, BMI, alcohol intake, and physical activity to examine the association between vitamin B2 intake and hepatocellular carcinoma risk (51). Vitamin B1 intake is negatively associated with the risk of colorectal cancer. A recent study showed no evidence of a correlation between vitamin B1 intake and the development of colorectal cancer (52). Liu’s team conducted a Meta-analysis to come to the same conclusion as ours: vitamin B1 intake is a protective factor in reducing the incidence of colorectal cancer (53). Animal experiments have shown that vitamin B1 deficiency may be associated with the development of colorectal cancer. They found an increased number of ACF (abnormal crypt foci) in the colon of rats fed a slightly vitamin B1-deficient diet (54). A randomized controlled trial found that plasma thiamine concentrations were generally below the reference range in patients with prior colon polyps or intramucosal cancer (55). In this study, vitamin E intake was negatively associated with the risk of hepatocellular carcinoma. Vitamin E prevents the production of N-nitroso compounds and is also a protective antioxidant against cancer. In previous studies, people taking vitamin E supplements (200 UI/day) may have a reduced risk of colon cancer (56). But we did not find that connection. More epidemiological studies are needed to confirm the relationship between vitamin E and digestive cancers.

We found a positive correlation between copper intake and gastric cancer in dietary intake. According to previous studies, copper concentrations were reported to be elevated in some types of cancers, including gynecological cancers, breast cancer, lung cancer, and gastrointestinal cancers (57–59). The relationship between copper and carcinogenesis appears well established, as cancer cells may require more copper than non-dividing cells (60). The results of this study suggest that dietary phosphorus has a protective effect against colorectal cancer. Some recently conducted studies indicate that phosphorus has a protective effect on the development of cervical intraepithelial neoplasia and colorectal adenomas, similar to our findings (61). Yumie reported that a combination of low calcium and high phosphorus may play a role in cancer development (62). However, previous studies have focused only on the dietary calcium-phosphorus ratio, not dietary phosphorus intake. Phosphorus intake only reflects daily intake but may not accurately reflect cell phosphorus levels (61). More epidemiologic studies are needed to verify the association between dietary phosphorus intake and digestive cancers. In a Mendelian randomization study examining the effect of micronutrient levels on the risk of colorectal polyps in humans, no statistically significant association was observed between 11 micronutrients, including calcium, and the risk of developing colorectal polyps (63). In another study, a low-calcium diet was found to be a diet-related risk factor for colorectal cancer (64). However, our study reached the opposite conclusion that calcium intake was positively associated with colorectal cancer and the development of liver cancer. Iron intake is positively associated with liver and colorectal cancer. Iron-induced oxidative stress leads to two possible consequences: (1) failure of redox regulation leading to lipid peroxidation and oxidative damage to DNA and proteins, and (2) activation of multiple mechanisms of reductive and hypoxic protection of redox regulation through signal transduction. Both outcomes appear to play a role in iron-induced carcinogenesis. In our study, zinc intake was positively associated with liver cancer. However, the study by Liu et al. concluded that both dietary zinc intake and serum zinc levels were negatively related to the risk of hepatocellular carcinoma, and the same conclusion was obtained even after adjusting for other risk factors for hepatocellular carcinoma, including hepatitis B virus infection, which is contrary to our findings (65).

This study also showed a positive association between protein intake and colorectal cancer. A recent study reported that a diet high in animal protein was associated with a moderate increase in the risk of colon cancer (66). The increased risk of colon cancer with a high intake of animal proteins may be due to their composition of red or processed meats, the consumption of which increases the risk of colon cancer (67). Our results showed that lycopene intake was positively associated with liver cancer. Previous studies have suggested that lycopene has a preventive effect against liver cancer. Still, Yin et al. reported that in a meta-analysis of the results of Mendelian randomization of different tumors, little or no reduction in the risk of liver cancer was observed with elevated levels of circulating lycopene (68).

It is important to emphasize that this study also has some limitations. First, it relied on a single measure of diet from a dietary recall interview. Each respondent’s dietary behavior may have changed over time, and there may also be some bias in the information recalled by some respondents. Second, some respondents may not have reported accurate information about their cancer history. Third, there may be complex additive effects and biological interactions between the various dietary categories in the usual diet (69). Although we have adjusted for known covariates, some other minor factors may influence the final conclusions we reach. Finally, in this study, the interviews were conducted after the respondents were diagnosed with cancer, so the respondents’ dietary information could have been changed after cancer treatment. Because this study was a cross-sectional study, it is challenging to elucidate the causal relationship between nutritional factors and digestive system cancers.

## 5 Conclusion

In conclusion, this study found a potential relationship between protein, vitamin B1, vitamin E, vitamin B12, vitamin B2, vitamin D, folate, copper, calcium, iron, phosphorus, zinc, lycopene intake and digestive system tumors. The results may provide insights into preventing tumors of the digestive system. Further epidemiological studies are needed to confirm these results.

## Data Availability

The datasets presented in this study can be found in online repositories. The names of the repository/repositories and accession number(s) can be found at: The database reported in this study is accessible via https://www.cdc.gov/nchs/nhanes/index.htm.
